# Blood Arsenic Levels as a Marker of Breast Cancer Risk among *BRCA1* Carriers

**DOI:** 10.3390/cancers13133345

**Published:** 2021-07-03

**Authors:** Wojciech Marciniak, Tomáš Matoušek, Susan Domchek, Angelo Paradiso, Margherita Patruno, Arvids Irmejs, Irita Roderte, Róża Derkacz, Piotr Baszuk, Magdalena Kuświk, Cezary Cybulski, Tomasz Huzarski, Jacek Gronwald, Tadeusz Dębniak, Michał Falco, Marcin R. Lener, Anna Jakubowska, Katherine Pullella, Joanne Kotsopoulos, Steven Narod, Jan Lubiński

**Affiliations:** 1 Department of Genetics and Pathology, Pomeranian Medical University, 71-252 Szczecin, Poland; wojciech.marciniak@read-gene.com (W.M.); roza.derkacz@read-gene.com (R.D.); piotr.baszuk@pum.edu.pl (P.B.); magdalena.kuswik@pum.edu.pl (M.K.); cezary.cybulski@pum.edu.pl (C.C.); huzarski@pum.edu.pl (T.H.); jgron@pum.edu.pl (J.G.); debniak@pum.edu.pl (T.D.); marcin.lener@pum.edu.pl (M.R.L.); aniaj@pum.edu.pl (A.J.); 2 Read-Gene SA, Westpomerania, 72-003 Grzepnica, Poland; 3 Institute of Analytical Chemistry of the Czech Academy of Sciences, 602 00 Brno, Czech Republic; matousek@biomed.cas.cz; 4 Basser Center for BRCA, Abramson Cancer Center, University of Pennsylvania, Philadelphia, PA 19104, USA; susan.domchek@pennmedicine.upenn.edu; 5 Center for Hereditary Tumors Research, Istituto Tumori Bari, Giovani Paolo II, IRCCS, 70124 Bari, Italy; av.paradiso@libero.it (A.P.); m.patruno@oncologico.bari.it (M.P.); 6 Department of Surgery, Institute of Oncology, Riga Stradins University, Pauls Stradins Clinical University Hospital, LV-1007 Rīga, Latvia; arvids.Irmejs@stradini.lv (A.I.); irita.roderte@rsu.lv (I.R.); 7 Department of Clinical Genetics and Pathology, University of Zielona Góra, 65-046 Zielona Góra, Poland; 8 West Pomeranian Oncology Center, Radiation Oncology Department, 71-730 Szczecin, Poland; falco.miw@op.pl; 9 Department of Nutritional Sciences, University of Toronto, Toronto, ON M5S 1A8, Canada; katherine.pullella@mail.utoronto.ca; 10 Dalla Lana School of Public Health, University of Toronto, Toronto, ON M5T 3M7, Canada; joanne.kotsopoulos@wchospital.ca; 11 Familial Breast Cancer Research Unit, Women’s College Research Institute, Toronto, ON M5G 1N8, Canada; steven.narod@wchospital.ca

**Keywords:** blood arsenic, cancer risk, epidemiology, *BRCA1* carriers, prospective cohort, breast cancer, cancer risk

## Abstract

**Simple Summary:**

Arsenic (As) is recognized by the International Agency for Research on Cancer (IARC) as a potent carcinogen. Numerous studies are focused on endemic regions of high exposure, and its effect on human health. Several authors suggest that As may be an important endocrine disruptor, mainly through estrogen-like activity. In our previous study on subjects without germline mutations in *BRCA1*, we reported that increased blood arsenic levels are significantly associated with high breast-cancer risk. The aim of this study was to assess if an association between As levels and cancer risk also exists among women harboring mutations in *BRCA1*. We found that women with As blood levels above the median (0.85 µg/L) had a significant 2-fold increased risk of developing breast cancer. This raises the possibility that lowering the blood arsenic level by dietary means might reduce cancer risk for *BRCA1* mutation carriers.

**Abstract:**

An important group of breast cancers is those associated with inherited susceptibility. In women, several predisposing mutations in genes involved in DNA repair have been discovered. Women with a germline pathogenic variant in *BRCA1* have a lifetime cancer risk of 70%. As part of a larger prospective study on heavy metals, our aim was to investigate if blood arsenic levels are associated with breast cancer risk among women with inherited *BRCA1* mutations. A total of 1084 participants with pathogenic variants in *BRCA1* were enrolled in this study. Subjects were followed from 2011 to 2020 (mean follow-up time: 3.75 years). During that time, 90 cancers were diagnosed, including 67 breast and 10 ovarian cancers. The group was stratified into two categories (lower and higher blood As levels), divided at the median (<0.85 µg/L and ≥0.85 µg/L) As level among all unaffected participants. Cox proportional hazards models were used to model the association between As levels and cancer incidence. A high blood As level (≥0.85 µg/L) was associated with a significantly increased risk of developing breast cancer (HR = 2.05; 95%CI: 1.18–3.56; *p* = 0.01) and of any cancer (HR = 1.73; 95%CI: 1.09–2.74; *p* = 0.02). These findings suggest a possible role of environmental arsenic in the development of cancers among women with germline pathogenic variants in *BRCA1*.

## 1. Introduction

Women with an inherited *BRCA1* mutation have a lifetime risk of developing breast cancer of approximately 70% [1,2,3]. Various lifestyle, dietary and environmental factors can contribute to an individual’s risk of developing breast cancer and provide an opportunity for intervention [2]. Over the past 50 years, there have been numerous studies focusing on the relationship between various components of the diet and cancer risk [4,5,6,7]. However, there is limited information regarding the relationship between dietary trace elements, such as arsenic, and cancer risk among *BRCA1* mutation carriers [8,9].

Arsenic is a metalloid that is widely distributed throughout the environment and can be found in water, soil and air [10]. Arsenic primarily exists in two forms: (1) inorganic arsenic, which has been deemed highly toxic, and (2) organic arsenic that is relatively non-toxic. Previous studies have shown that exposure to arsenic has been linked to harmful health outcomes, including the development of neurological disorders, cardiovascular disease and cancers [11,12,13,14]. The International Agency for Research on Cancer (IARC) has categorized inorganic arsenic as a bona fide carcinogen for cancers of the lung, skin and bladder; however, the relationship between arsenic levels and breast cancer risk is not clear [15]. To date, previous studies focusing on cancer risk evaluation have been conducted in areas where high arsenic exposure is endemic [16]. In these populations, the levels of arsenic reported in water supplies often exceed levels that are observed in European countries [17,18].

We have recently demonstrated a significant association between total blood arsenic levels and the risk of breast cancer in a population of Polish women who did not carry a *BRCA1* mutation. We reported that women in the highest quartile of total blood arsenic had a 13-fold increased risk of breast cancer, compared to women in the reference group [19]. To date, little is known about the impact of chronic exposure to low levels of arsenic in women who have an inherited susceptibility to breast cancer [19]. In the present study, we prospectively evaluate the relationship between total arsenic levels, measured in whole blood, and breast cancer risk among women carrying mutations in *BRCA1*.

## 2. Materials and Methods

### 2.1. Study Subjects

The study subjects were women aged 25 years and above, who received genetic counselling and testing between January 2011 and August 2020. All participants had no prior history of breast, or other, cancers at the time of enrollment in the study. All participants provided informed, written consent to participate in the study, and agreed to provide a blood sample to be used for research purposes [19]. The study cohort was comprised of individuals from a multi-centre collaboration between Pomeranian Medical University in Szczecin, Poland, Pauls Stradins Clinical University Hospital in Latvia and the Clinical Experimental Oncology Laboratory in Italy. The study protocol was approved by the Pomeranian Medical University research ethics board.

At the first study visit, a fasting blood sample was collected from each study participant to be used for genetic testing for *BRCA1* mutations. Additionally, a second aliquot of 2 cc of whole blood was collected for research purposes. All blood samples were collected between 8 a.m. and 2 p.m. and stored at −80 ℃ until analysis. Participants were included in the study if a deleterious *BRCA1* variant was detected.

Women without *BRCA1* deleterious mutations were excluded from this study, and results from that sub-cohort have been reported in a prior publication [19].

Study participants also completed a detailed questionnaire regarding their age, prior contraceptive and hormone replacement therapy use, smoking habits, personal medical history, including ovary removal surgery (oophorectomy), use of dietary supplements, diabetes, and body mass index (BMI).

### 2.2. Total Arsenic Determination

Total blood arsenic was quantitatively measured using inductively coupled plasma mass spectrometry, using the ELAN DRC-e instrument (PerkinElmer, Norwalk, CT, USA). Arsenic was measured under the DRC (Dynamic Reaction Cell) conditions with oxygen as a reaction gas (O_2_ purity > 0.9999, Messer). Samples were diluted 30 times using an alkali buffer. For best accuracy, matrix-matched technique was used for calibration. The calibration curve consisted of 0.0 µg/L; 0.48 µg/L; 0.96 µg/L; 1.98 µg/L and 2.0 µg/L calibration points, for which the linear coefficient was always greater than >0.999. Rhodium was set as an internal standard to compensate instrument drift and matrix effects. Parameters of the method were confirmed by using several available certified materials: NIST 955C (NIST, USA), Plasmonorm Whole Blood Level 1 (ClinCheck, Recipe, Germany).

Additionally, blood lead and cadmium levels were measured as described [20] in order to assess possible interactions.

### 2.3. Statistics

There were 1084 women enrolled in this study. All were free of cancer at baseline and carried a mutation in the *BRCA1* gene. Women were followed from the date of blood draw or age 25 years (whichever came last) to the date of first breast cancer diagnosis, death from another cause, or 18 August 2020.

Information on diagnosed cancers was extracted from the medical records and manual review of pathology records from the treating hospitals in Szczecin (Poland), Bari (Italy) and Riga (Latvia). Study participants were assigned to one of two categories (lower and higher blood As levels) divided by the median (<0.85 µg/L and ≥0.85 µg/L) As level among all unaffected participants. In order to estimate the hazard ratio (HR) for breast cancer risk, univariable and multivariable Cox proportional hazards regression models were calculated. In multivariable models, the following factors were taken into analysis: arsenic median, age at blood draw (≤50 vs. >50), oral contraceptive use (ever/never), hormone replacement therapy use (ever/never), smoking history (yes/no), oophorectomy (yes/no), dietary supplement use (ever/never), diabetes (yes/no), BMI (<18.5; 18.5–24.9; 25.0–29.9; ≥30.00). BMI was stratified to four categories: <18.5 as underweight, 18.5–24.9 as normal weight; 25.0–29.9 as pre-obesity; ≥30.00 as obesity. To exclude the potential influence of other toxic metals on breast cancer risk, levels of lead and cadmium were included in the multivariable analysis. Subjects were assigned to one of two categories (lower and higher) divided by the median (11.75 µg/L for Pb and 0.42 µg/L for Cd) level among all unaffected participants.

Cancer-free observation time (or time to diagnosis) varied between 0 and 8 years. Crude differences in cumulative rate incidence by the As level were tested for statistical significance using the log-rank test. A secondary analysis was conducted using the diagnosis of any cancer as the outcome. For this analysis, all patients were followed from date of blood draw or age 25 years (whichever came last) to the date of the first cancer diagnosis, death from another cause or 18 August 2020.

All statistical analyses were performed using R statistical environment (R version 4.0.3 (2020-10-10); Copyright (C) 2020 The R Foundation for Statistical Computing).

## 3. Results

This study included 1084 women (1022 from Poland, 48 from Italy and 14 from Latvia). All were positive for a deleterious variant in *BRCA1*. A total of 46 different *BRCA1* deleterious variants were detected in this cohort, and three founder mutations (c.5266dupC-5382insC; c.181T>G-300T>G; c.4035delA-4153delA) contributed 89.39% of all detected variants. All study participants had no prior history of cancer at the time of enrollment in the study. Total blood arsenic levels for all women in the cohort are presented in Table 1. Mean age at the time of blood draw was 41.77 years (range 25–75).

The women were followed for an average 3.75 years (range 0–8 years) from the blood draw. Over the follow-up period, 90 incident cases of cancers were diagnosed in the study cohort (Table 2).

This included 67 cases of breast cancer, 10 cases of ovarian cancer, and 13 cancers at other sites. One study participant was affected by both breast and ovarian cancer during the follow-up period. For analysis, the date and type of the first cancer was used.

The 1084 study participants were assigned to one of two categories (lower and higher blood As levels) divided by the median (<0.85 µg/L and ≥0.85 µg/L) As level among all unaffected participants. The univariate and multivariate hazard ratios (HR) for developing breast cancer, stratified by median blood arsenic levels, are presented in Table 3.

In the crude analysis, a higher level of total blood arsenic (≥0.85 µg/L) was associated with a significantly increased risk of developing breast cancer (HR = 2.03; 95% CI: 1.18–3.49, *p* = 0.011). The effect was similar after adjusting for age at blood draw (≤50 vs. >50), oral contraceptive use (ever/never), hormone replacement therapy use (ever/never), smoking history (yes/no), oophorectomy (yes/no), dietary supplement use (ever/never), diabetes (yes/no), BMI (<18.5; 18.5–24.9; 25.0–29.9; ≥30.00), levels of other toxic metals, such as lead and cadmium (HR = 2.05; 95% CI: 1.18–3.56, *p* = 0.011).

**Figure 1:**
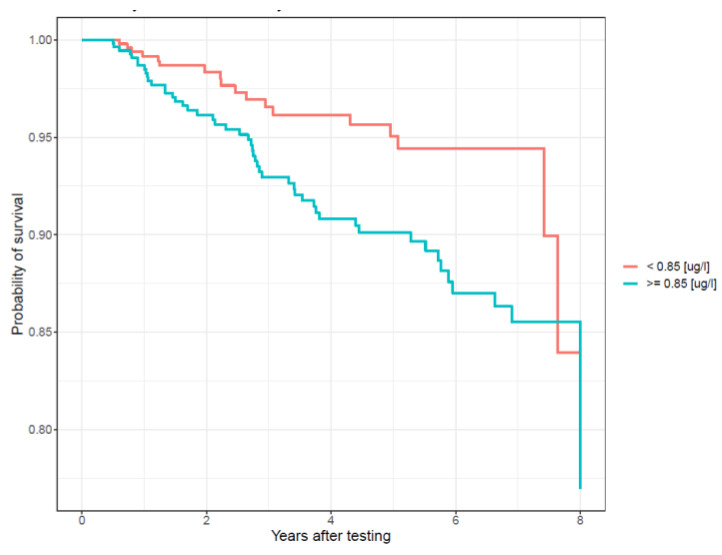
Probability of breast cancer-free survival stratified by median total blood arsenic levels (µg/L).

After 5 years of follow-up, the cumulative incidence of breast cancer was estimated to be 6% for the low blood arsenic group (<0.85 µg/L) and 10% for the high blood arsenic group (≥0.85 µg/L) (Figure 1).

In a secondary analysis, the diagnosis of any cancer was considered as the endpoint (*n* = 90). Similar to the previous analysis, women were assigned to one of two categories (lower and higher blood As levels) divided by the median (<0.85 µg/L and ≥0.85 µg/L) As level among all unaffected participants. The univariate and multivariate hazard ratios are presented in Table 4.

Overall, women who had higher total blood arsenic levels (≥0.85 µg/L) had a 1.7 increased risk of developing any cancer, compared to the lower blood arsenic reference group (adjusted HR = 1.73, 95%CI: 1.09–2.74, *p* = 0.02).

**Figure 2:**
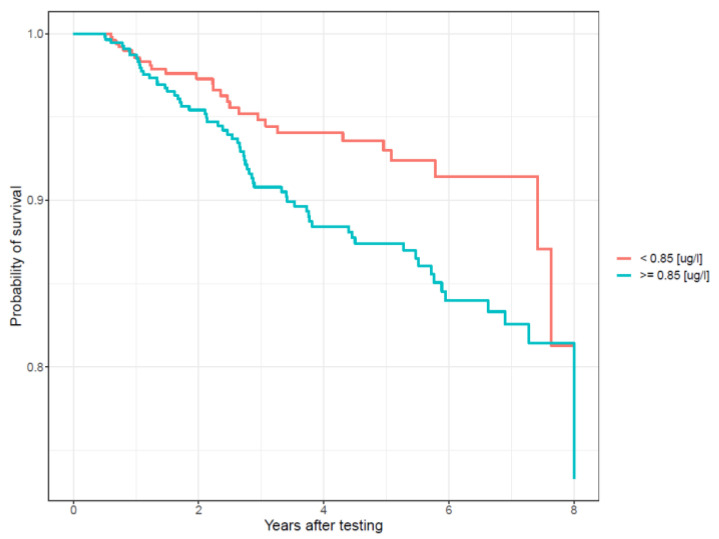
Probability of cancer-free survival stratified by median total blood arsenic levels (µg/L).

After 5 years of follow up, the cumulative incidence of cancer was 7% for the low total blood arsenic group (<0.85 µg/L) and 13% for the high total blood arsenic group (≥0.85 µg/L) (Figure 2).

## 4. Discussion

In this prospective study of 1084 European women, we evaluated the impact of arsenic exposure on individuals who have inherited susceptibility to breast cancer because of mutations in the *BRCA1* gene. The findings of this study revealed a statistically significant association between baseline blood arsenic levels and subsequent risk of breast cancer, and with all cancers combined. Women with a higher total blood arsenic level (≥0.85 µg/L) had a 2.05 increased risk of developing breast cancer, compared to the lower blood arsenic reference group (*p* = 0.011). The risk ratio was not modified by adjusting for potential confounders.

Similarly, in an analysis of all cancers combined, the HR comparing women in the higher blood arsenic group to the lower reference group was 1.73 (*p* = 0.02). When comparing these findings to those of our previous study in women who did not have a *BRCA1* mutation [19], this observation strengthens the evidence suggesting that exposure to low levels of arsenic may have an impact on cancer incidence in women.

Exposure to arsenic occurs primarily through consumption of various foods and beverages, especially in low-exposure populations [21]. Such items include water, rice, pitted fruits, various vegetables, fish and seafood. Due to the ubiquitous nature of the element, exposure to low levels of arsenic occurs daily, and overall levels of exposure can be quantified in one of four biological specimens: blood, urine, hair and nail samples. Each sample is reflective of a distinct biological burden and is suggestive of a different timeframe of exposure [16].

There are several studies about the influence of blood As on disease risk in different populations [22]. Most of the papers come from countries that are located in areas known to be polluted by high amounts of As. Additionally, it is hard to compare the results from the Polish population with, e.g., Japanese people, who have completely different dietary habits (i.e., seafood consumption) [22]. There is one paper on blood As levels in Polish women [19]. In the previous study, the end of second quartile (median As level) was 0.82 µg/L, extremely close to current results.

The total blood arsenic concentrations, as measured in this study, quantifies exposure to both inorganic and organic forms of arsenic [21,23]. Study subjects are consuming foods naturally enriched in non-toxic organic arsenic, which is the prominent arsenic form in fish and seafood. The dietary sources of arsenic will be investigated in a subsequent study.

Once ingested, the As is mainly absorbed through the gastrointestinal tract [24]. All arsenic species are rapidly taken up into the bloodstream and widely distributed in tissues. The mobility of As is strongly dependent on its form. Inorganic arsenicals are more easily absorbed than organic derivatives [25]. Arsenicals enter the cell through the aquaglyceroporins using the structural similarity to phosphate compounds [26]. Due to the affinity of inorganic species of arsenic to sulfhydryl groups, As can be found in keratin-rich tissues, such as skin, hair and nails, for a long time after ingestion. Higher amounts of arsenic were found in tissues of lung, urinary bladder, kidney and liver [25].

The methylation of arsenic is thought to be one of the primary aspects of the element’s carcinogenicity. Currently accepted mechanisms suggest that incomplete methylation of inorganic arsenic to dimethylarsinic acid leads to the bioaccumulation of toxic arsenic species (such as MMA^III^, DMA^III^) in the body [27,28]. Such investigations should be included in future studies of arsenic exposure and breast cancer risk in high-risk populations, in order to develop specific recommendations.

The observed weaker effect of arsenic exposure on cancer risk in *BRCA1* mutation carriers, compared to our earlier work on those who did not inherit mutations, may be due to a difference in mean age at the blood draw. In this cohort of *BRCA1* mutation carriers, the average age of study participants was 41.4 years at the time of blood draw, in comparison to 55.2 years at the time of blood draw in the cohort of women without any observed mutation. A study conducted by Smith et al. in 2018 suggested that there could be a latent response between exposure to arsenic and occurrence of malignancies, which might not be accurately captured in this younger cohort [29].

There is a growing body of evidence suggesting a relationship between low levels of arsenic and breast cancer risk; however, the mechanism by which low levels of arsenic exposure increase this risk remains unclear. Previous work has elucidated that sodium arsenite mimics the effects of estradiol and can lead to induction of cell proliferation, migration and invasion, suggesting that inorganic arsenic can act as an endocrine disruptor [30]. Liu et al. (2015) reported that long-term exposure to low levels of arsenic in ambient air is significantly associated with an increased risk of developing hormone-receptor-negative breast cancers (ER−, PR−, HER−), the predominant form among *BRCA1* mutation carriers [31,32]. Given that Poland, Italy and Latvia are located in the regions where arsenic contamination in drinking water is not a concern, dietary exposure remains regarded as the primary route of exposure for these populations [33,34]. However, exposure to airborne arsenic will be evaluated in future studies [32].

We cannot exclude the possibility that arsenic might be only a marker of foods that are actually enriched in other carcinogens. Future studies should evaluate the overall impact of exposure to a mixture of several toxic heavy metals that are known to co-exist, such as mercury, lead and cadmium, so as to clearly delineate this relationship. Additionally, future work should be focused on potential modifiers of arsenic exposure (i.e., polymorphisms of genes involved in xenobiotics metabolism), as well as genome methylation. Validation studies on additional cohorts and from different populations are also needed.

## 5. Conclusions

Blood arsenic level is the marker of cancer risk among *BRCA1* mutation carriers. This correlation is attractive as it creates the perspective that lowering blood arsenic level by control of diet can significantly improve the situation of carriers. 

## Figures and Tables

**Table 1:** Characteristics of 1084 women in the cohort.

Characteristics	*n* (%)	Mean Arsenic Level µg/L (range)
Age		
≤50	831 (77)	1.29 (0.26–70.4)
>50	253 (23)	1.41 (0.33–13.7)
Contraceptive Usage		
Ever	478 (44)	1.34 (0.27–17.46)
Never	606 (56)	1.30 (0.26–70.04)
Hormonal Replacement Therapy Usage		
Ever	167 (15)	1.81 (0.26–70.04)
Never	917 (85)	1.23 (0.26–17.46)
Smoking Status		
Yes	341 (31)	1.23 (0.26–17.46)
No	743 (69)	1.36 (0.26–70.04)
Oophorectomy		
Yes	510 (47)	1.42 (0.26–70.04)
No	574 (53)	1.23 (0.26–12.97)
Dietary Supplements Usage		
Ever	191 (18)	1.18 (0.26–9.65)
Never	893 (82)	1.35 (0.26–70.04)
Diabetes		
Yes	34 (3)	1.60 (0.40–6.94)
No	1050 (97)	1.31 (0.26–70.04)
Body Mass Index (BMI)		
<18.5	67 (6.2)	1.17 (0,29–7.18)
18.5–24.9	649 (60)	1.32 (0.27–70.04)
25.0–29.9	263 (24)	1.24 (0.26–13.70)
≥30.0	105 (9.8)	1.60 (0.34–12.37)
Blood Lead Level		
<11.75	537 (49.34)	1.19 (0.26–12.97)
≥11.75	547 (50.66)	1.45 (0.26–70.04)
Blood Cadmium Level		
<0.42	528 (49.96)	1.21 (0.26–12.97)
≥0.42	556 (50.04)	1.43 (0.25–70.04)

**Table 2:** Incident cancers detected in the cohort (*n* = 90).

Cancer Site	*n*	Mean Blood Arsenic Level µg/L (range)
Breast	67	1.65 (0.39–17.46)
Ovarian	10	1.33 (0.38–2.33)
Cervix	3	1.28 (0.60–2.26)
Peritoneal	2	0.88 (0.59–1.17)
Colon and intestine	1	0.36
Bladder	1	0.63
Kidney	1	1.4
Larynx	1	0.98
Leukemia	1	1.06
Skin	1	1.39
Stomach	1	0.7
Thyroid	1	0.85

**Table 3:** Hazard ratios (HR) for breast cancer stratified by the median total blood arsenic level (µg/L) *n* = 1061.

Arsenic Level (µg/L)	Total	Cancers	Univariate HR (95%CI)	*p*	Multivariate HR * (95%CI)	*p*
<0.85	513	18	1 (Ref.)	-	1 (Ref.)	-
≥0.85	548	49	2.03 (1.18–3.49)	0.011	2.05 (1.18–3.56)	0.011

* HR adjusted age at blood draw ( ≤50 vs. >50), oral contraceptive use (ever/never), hormone replacement therapy usage (ever/never), smoking history (yes/no), oophorectomy (yes/no), supplement use (ever/never), diabetes (yes/no), BMI (<18.5; 18.5–24.9; 25.0–29.9; ≥30.00), levels of other toxic metals, such as lead and cadmium (levels divided into 2 categories in the same manner as for arsenic).

**Table 4:** Hazard ratios (HR) for any cancer stratified by median total blood arsenic level (µg/L) = 1084.

Arsenic Level (µg/L)	Total	Cancers	Univariate HR (95%CI)	*p*	Multivariate HR * (95%CI)	*p*
<0.85	522	27	1 (Ref.)	-	1 (Ref.)	-
≥0.85	562	63	1.73 (1.10–2.71)	0.01	1.73 (1.09–2.74)	0.02

* HR adjusted age at blood draw (≤50 vs. >50), oral contraceptive use (ever/never), hormone replacement therapy usage (ever/never), smoking history (current/past/never), oophorectomy (yes/no), supplement use (ever/never), diabetes (yes/no), BMI (<18.5; 18.5–24.9; 25.0–29.9; ≥30.00), levels of other toxic metals such as lead and cadmium (levels divided into 2 categories in the same manner as for arsenic).

## Data Availability

The data presented in this study are available on request from the corresponding author.
